# A case report of a giant ileocecal cystic prolapse through the anus and literature review

**DOI:** 10.3389/fmed.2023.1324792

**Published:** 2024-01-05

**Authors:** Beige Zong, Xia Xiao, Nijiao Deng, Wenjing Wang, Li Peng, Dianliang Fang, Haoyu Wang, Song Hu, Zhongfu Li, Xin Zhang

**Affiliations:** ^1^Department of General Surgery, The Fourth People’s Hospital of Chongqing, Chongqing University Central Hospital, Chongqing Emergency Medical Center, Chongqing, China; ^2^Medical Imaging Department, The Fourth People’s Hospital of Chongqing, Chongqing University Central Hospital, Chongqing Emergency Medical Center, Chongqing, China; ^3^Department of Pathology, The Fourth People’s Hospital of Chongqing, Chongqing University Central Hospital, Chongqing Emergency Medical Center, Chongqing, China; ^4^Department of Gastroenterology and Hepatology, The Fourth People’s Hospital of Chongqing, Chongqing University Central Hospital, Chongqing Emergency Medical Center, Chongqing, China

**Keywords:** enterogenous cyst, intestinal duplication, exploratory laparotomy, intussusception, acute abdominal disease

## Abstract

Intussusception refers to the invagination of a proximal loop of the bowel into an adjacent distal segment. This condition is rare in adults, especially when it involves a complete folding of the ileocecal area out of the body cavity. Meanwhile, enterogenous cysts are congenital malformations that are largely identified in childhood following symptoms of bowel obstruction. While surgical treatment is ultimately required for both diseases, deciding on the type of surgery and the right time to operate can be a challenge for clinicians. It is especially difficult to decide on treatment for an adult with the coincidental occurrence of both conditions and no definitive pathologic diagnosis prior to surgery. Here, we present the case study of a 19-year-old female patient who presented with a prolapsed anus due to intussusception caused by a large ileocecal mass. The patient was admitted to the emergency department with a “massive anal mass.” She remained symptomatic after receiving conventional conservative treatment and had to undergo emergency surgery after developing an intestinal obstruction. While the patient’s intraoperative condition also confirmed the preoperative CT findings, the situation became more complicated during surgery. The postoperative pathological report indicated the presence of an enterogenous cyst. After recovery from surgery, the patient was successfully discharged. Intussusception or intestinal obstruction caused by an intestinal mass is a surgical indication, and removal is the only way to cure the condition. This case study provides a helpful reference for general surgeons, especially anorectal surgeons, imaging physicians, and pathologists, and informs the diagnosis and treatment of this patient population.

## 1 Introduction

Intussusception is the invagination of a proximal loop of the bowel into an adjacent distal segment. The most common sites for intussusception include the cecum, transverse colon, and sigmoid colon with unfixed peritoneum. While this condition is common in very young children and the elderly (1, 2), it is rare in adults, especially when it involves complete folding of the ileocecal area out of the body cavity. When an external anal mass occurs, conventional diagnoses and treatment plans often consider perianal or rectal prolapse diseases (3). Meanwhile, enterogenous cysts are a congenital malformation that is mostly found in childhood following symptoms of bowel obstruction (1, 4). The incidence of this disease has not been reported because it is mostly described in individual case reports. While the treatment of both diseases is ultimately dependent on surgery (5, 6), choosing the type of surgery and the optimal time to operate can be challenging. Laparoscopic exploration is the first choice for the diagnosis and treatment of internal abdominal diseases (2); however, this is most appropriate for patients with severe gas obstruction and unknown abdominal conditions (7).

Enterogenous cysts are congenital abnormalities that are very rare in adults and adult intussusception caused by these cysts is identified even less frequently. Enterogenous cysts resulting from folding of the ileocecal area along the intestinal cavity and out of the anus have never been reported. Our department recently admitted such a patient after recovery from surgical treatment.

## 2 Case presentation

A 19-year-old female patient (165 cm, 45.1 kg, BMI = 16.6) was hospitalized in September 2023 after presenting with “abdominal pain for 2 months with huge anal mass protrusion for 9 h.” Approximately 2 months before admission, the patient experienced abdominal pain without an obvious cause and had occasional colic that was accompanied by anal distension. She had been treated for “gastroenteritis” with oral “anti-inflammatory drugs” and “probiotics” in other hospitals. The pain was slightly relieved, and the patient did not receive further treatment. Nine hours before admission, the patient felt the anal mass coming out while she was going to the toilet, experienced lower abdominal pain, and was sent to the emergency department. She and her family denied any prior episodes of similar symptoms. After admission, the patient was diagnosed with anal mass prolapse for investigation as potential rectal prolapse, a malignant tumor, or other cause. A physical examination of the patient revealed that she had a temperature of 36.5°C, a heart rate of 76 beats/min, a respiratory rate of 20 breaths/min, an oxygen saturation (SpO2) of 99%, and a blood pressure of 112/76 mmHg. She experienced abdominal tenderness, mainly around the umbilicus and lower abdomen, and could not accurately describe the location and nature of her abdominal pain. A red mass of approximately 100*60*60 mm in size was visualized outside the anus. The surface was backed by red mucous membranes, and the interior appeared to touch a tough mass with clear boundaries. Intestinal wall-like wrinkled tissue was present near and connected to the anus. Other physical tests were negative and a blood test indicated a white blood cell (WBC) count of 11.26 × 10^9^/L.

**Figure 1:**
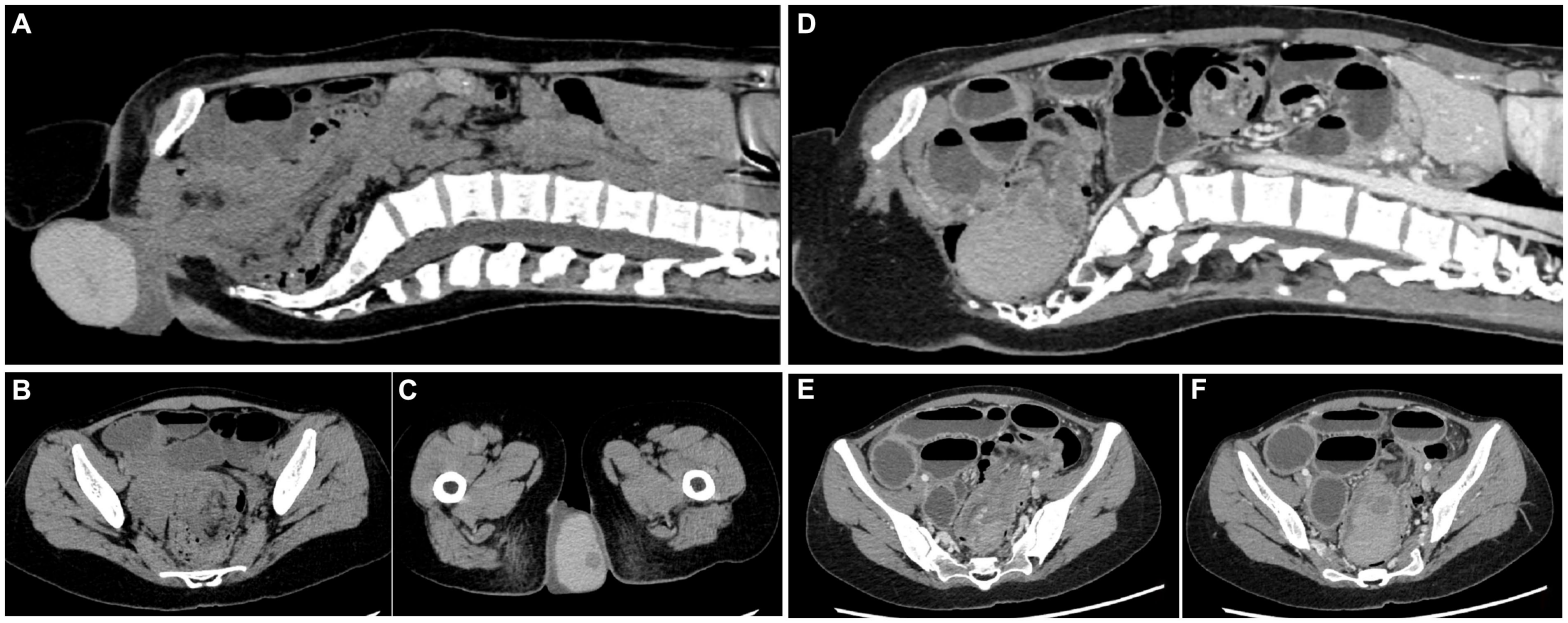
Computed tomography of the abdomen and pelvis. **(A–C)** Plain CT before the return of the cyst. **(D–F)** Enhanced CT after the return of the cyst. The intestinal duct structure is disordered, and the small intestine is obviously dilated.

Upon admission, an abdomen and pelvis computed tomography (CT) scan indicated an intestinal structure disorder combined with incomplete intestinal obstruction, suspicious intussusception, and partial intestinal duct prolapse through the anus. An external anal mass high-density shadow (CT value ≈ 90) of size ≈ 78*61*41 mm and nature unknown was identified (Figures 1A–C). Based on these findings, the root of the mass was anticipated to be at least above the junction of the rectosigmoid colon.

After the initial exclusion of an anal mass and rectal prolapse, the potential for damage to the patient by emergency surgery without a clear diagnosis was considered. The patient felt abdominal pain relief when the mass was reinserted into the anus by manipulation. Treatment included fasting, relief of intestinal spasms, fluids, and antibiotics. After the edema of the mass subsided, further examination using colonoscopy and enhanced CT was considered to determine the nature and location of the mass prior to further treatment. During the observation period, the patient reported exhaust gas and no defecation. A physical examination of the abdomen showed no obvious mass and slightly hyperactive bowel ringing (5–6 times/min).

To further determine the nature of the mass and its relationship with the intestine, a colonoscopy was performed 2 days later. The patient was orally prepared with polyethylene glycol electrolyte powder 42 h after the mass was restored (8). Within 3 h, the intake was approximately 1,300 mL and the vomiting was approximately 500 mL, largely including polyethylene glycol electrolyte powder liquid containing gastric juice. After the mass had returned for 54 h (48 h + 6 h), the patient underwent a colonoscopy under intravenous anesthesia. Screening revealed a large smooth-surfaced mucosal mass in the rectum. The mass had extended into the descending colon, which contained a large amount of feces that could not be passed. Intussusception was considered highly probable. After communication with the patient and her family members, the patient underwent an enhanced abdominal CT. A pelvic cystic mass with clear boundaries and thicker, uniform internal density was identified. There was no enhancement in the cyst, but obvious enhancement in the cyst wall that was consistent with that of the surrounding intestinal duct. The findings indicated that the ileocolecular intussusception was associated with a low-level intestinal obstruction that had become aggravated since the patient’s initial hospitalization on 16 September. A large, dense mass at the distal end of the intussusception was located in the pelvic cavity, and a cystic lesion was considered (Figure 1).

**Figure 2:**
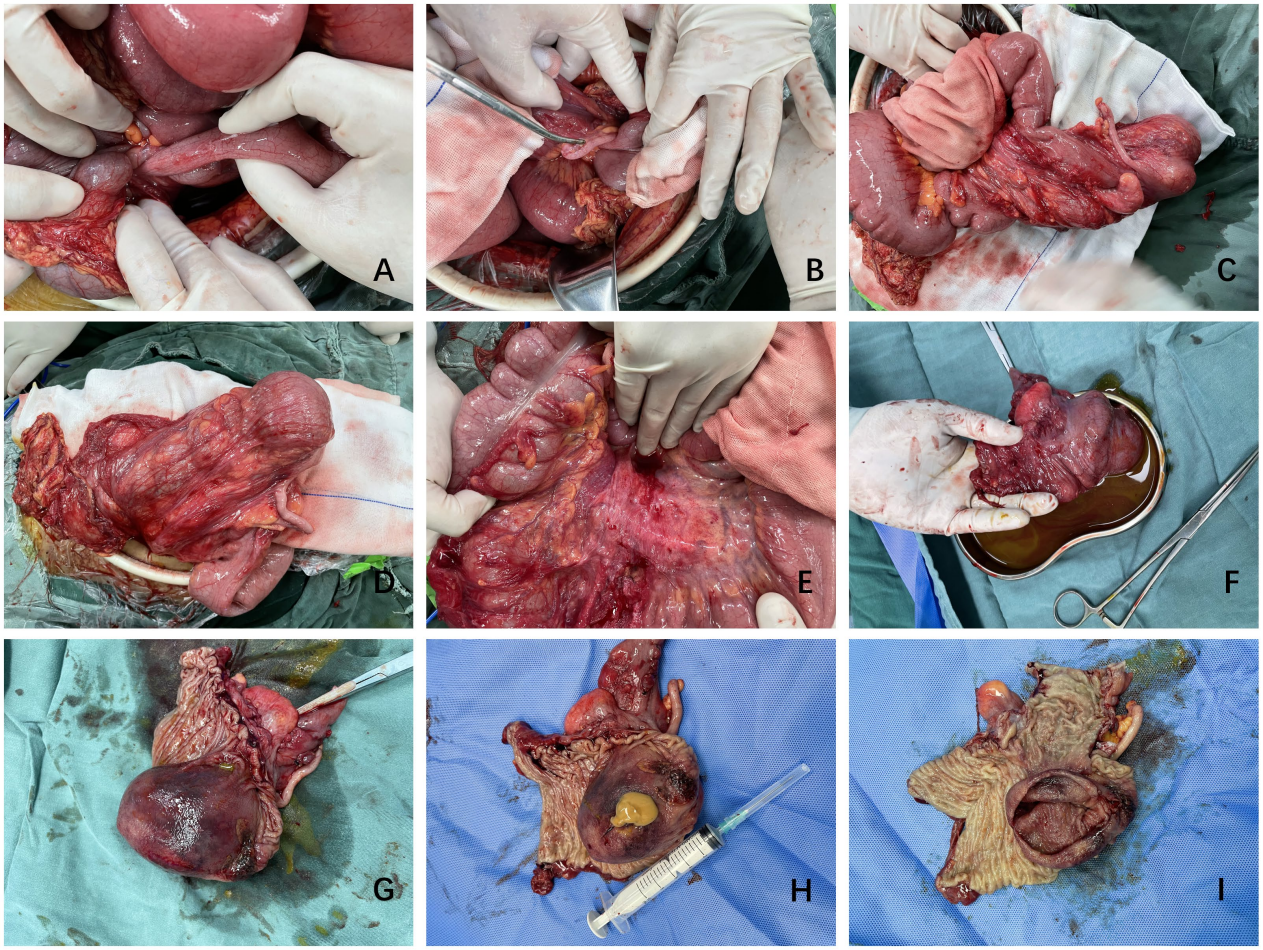
Representative surgical images. **(A–E)** Images from various stages of the operation showing the overlap of the small intestine and colon. The colon is free and not fixed and the cyst is located in the ileocecal region. **(F–I)** Postoperative cyst specimen showing the smooth cyst wall and presence of fluid. The cyst is located in the ileocecal region.

The presence of intussusception with intestinal obstruction indicated that emergency surgery had to be performed. After communicating with the patient and her family about the necessity of surgical treatment, the patient signed the relevant surgical informed consent. She underwent ileocecal resection and colo-small intestine anastomosis under general anesthesia on 20 September 2023. During the operation, the proximal part of the small intestine was significantly dilated, and the distal part was nested and intertwined with the colon (Figure 2A). The mass could be touched in the descending colon. The ileum was carefully pulled while pressing the mass from the distal end. This was the most important and difficult step of the operation. After revealing the exposed appendix (Figure 2B), the compressed and overturned ileocecal mass fully exposed the ileocecal region. The patient’s entire colon was treated in a free state and placed completely outside the abdominal cavity (Figures 2C,D). Fibrous, thickened, and enlarged lymph nodes were revealed within the mesentery (Figure 2E).

Postoperatively, the cyst could be seen formed part of the ileocecal region and was almost protruding from the intestinal lumen (Figures 2F,G). The surface of the cyst was damaged by the friction caused by prolapse outside the anus (Figures 1, 2G). When the capsule wall was cut open, a yellow viscous fluid flowed out. After wiping the posterior capsule fluid, the intact capsule wall was observed (Figures 2H,I). The cut cyst was soaked in formalin to prepare it for pathological biopsy.

The patient was treated and cared for according to the principle of enhanced recovery after surgery.

On the second day after surgery, the patient got out of bed and walked after passing flatus. Three days after surgery, the patient was told to drink a little water and after 5 days, the patient began a light liquid diet. The patient recovered and was discharged from the hospital 8 days after surgery once the abdominal drain was removed.

**Figure 3:**
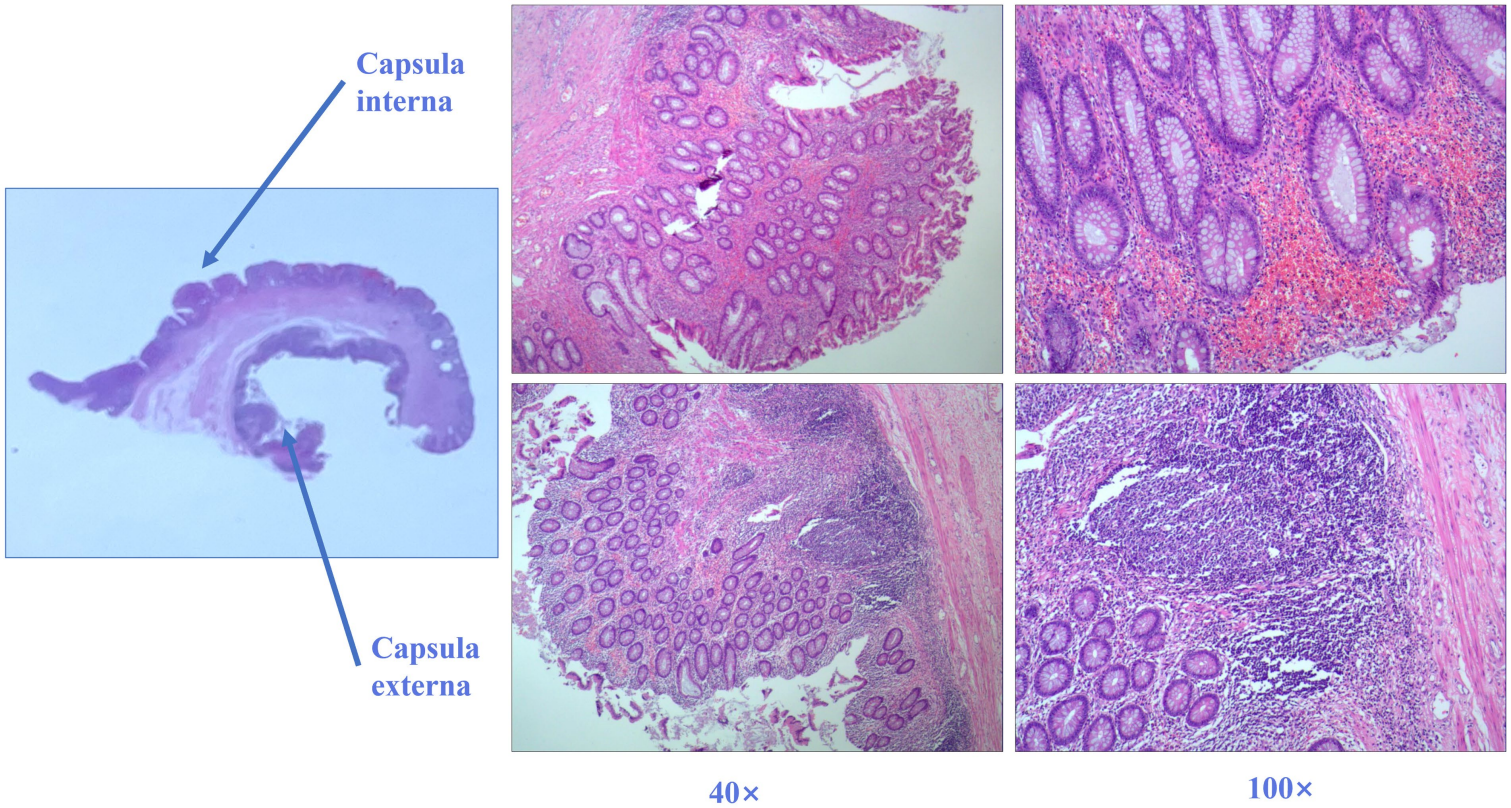
Postoperative histology of the ileocecal mass. Hematoxylin and eosin-stained sections of the ileocecal mass and its link to the intestine show evidence of the enterogenous cyst. The intestinal epithelium covers the bilayer capsule wall (40× and 100× magnification).

The postoperative pathological results indicated the presence of an enterogenous cyst. Microscopically, the mass was cystic and the muscle wall structure was visible in both layers of the cyst wall. The inner capsule was lined with intestinal epithelial mucosa, and there was obvious lymphoid tissue hyperplasia that resembled the structure of the appendix. An external capsule was attached to the intestinal epithelial mucosa and interstitial bleeding, inflammatory cell infiltration, and a small amount of necrosis were observed (Figure 3).

The final emergency surgery relieved the patient of the intestinal obstruction caused by intussusception. The ileocecal area containing the diseased tissue was excised, preventing the formation of an abdominal stoma.

**Figure 4:**
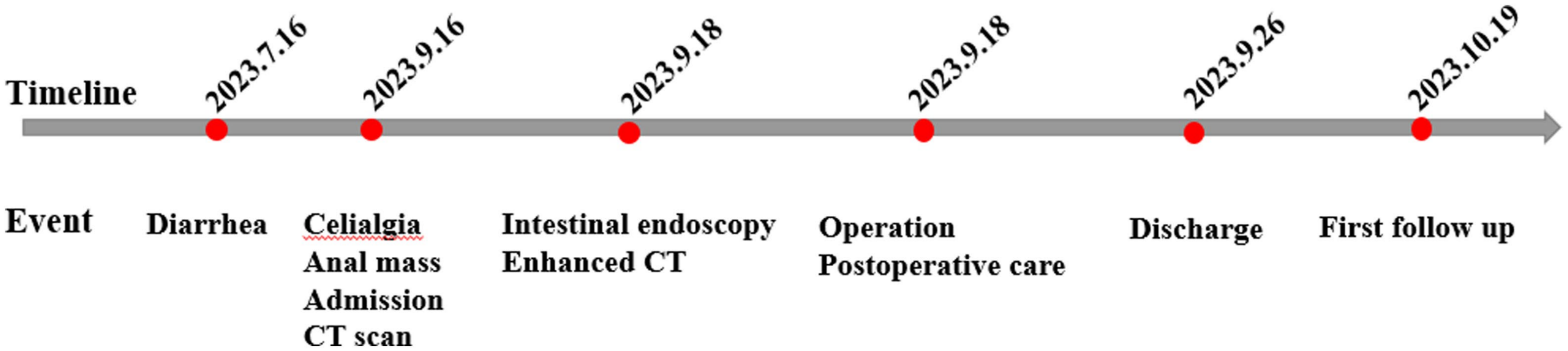
Timeline of the patient’s treatment scheme.

One month after surgery, the patient was followed up by telephone and asked questions relating to her diet, stool color and character, occurrence of abdominal pain and constipation, and any problems with sleep or mental state. The patient reported that she had basically returned to a normal life without symptoms of abdominal discomfort. She was told to come to the outpatient clinic for follow-up after 3 months. The entire treatment process of this patient is summarized in the flowchart in Figure 4.

## 3 Discussion

Enterogenous cysts are rare congenital malformations that are defined by the World Health Organization as “lined with mucus-secreting epithelium similar to that of the gastrointestinal tract” (5). Enterogenous cysts, also known as enteric duplication, are identical cystic tissues found along the adjacent intestinal wall at the mesangial margin of the digestive tract (1). The enteric duplication subtype accounts for approximately 80% of these cysts and their appearance is round or oval. There is a significant difference between the cyst cavity and the intestinal duct (9). While the case reported here had the enteric duplication subtype, there was little histological difference between the inner and outer walls of the cyst (Figure 3).

The pathogenesis of enterogenous cysts remains unclear, and most researchers believe that it is caused by a blurred embryonic primordium that occurs during the development of tissues and embryos. Approximately a third of cases are complicated by other malformations, such as intestinal atresia, omphalocele, and intestinal malrotation (10, 11). The mechanism for the development of these cysts remains unclear and requires further exploration by embryologists and pathologists.

Enterogenous cysts can occur at any age but are more common in children (1, 4). The disease occurs mostly in the spinal canal, intracranial and mediastinum (6), retroperitoneum (4), and digestive tract in the small intestine (5), but is rare in the ileocecal region (12, 13). This case study describes a patient with a large enterogenous cyst located in the ileocecal region and found outside the anus. The intraoperative findings suggested that the patient was complicated with congenital mesocolon dysplasia. Due to the larger volume and mobility of the abdominal cavity as compared to the spinal canal or the skull, the patient grew into a healthy adult without any discomfort caused by this cyst. In later years, as a result of diarrhea and other undefined issues, intestinal peristalsis caused the ileocecal mass to entangle into the colon and prostrate out of the anus along the entire colon duct. This has not been reported in previous case reports.

The following conclusions can be summarized from this case:

Prolapse of an anal mass is a common symptom of anorectal diseases. Upon observation of an anal mass prolapse, a clinician’s first consideration may be large hemorrhoids, rectal prolapse (3), or a rare sigmoid colon mass prolapse from the anus (14, 15). The current case report describes a young female patient with no history of hemorrhoids, fertility, or abdominal trauma. After a physical examination and an assessment of the mass, it was speculated that the mass originated from the upper rectal segment. We were surprised to observe that the mass originated from the ileocecal region. Considering the harm that could be caused by persistent intussusception and intestinal obstruction, emergency surgery was performed. This method is consistent with the treatment principle that complete removal of the enterogenous cyst wall is the key to surgical cure (1). While bowel preparation had the potential to aggravate the symptoms of intestinal obstruction during treatment, the patient’s vital signs were stable and she had no symptoms of intestinal obstruction. It is important to avoid intestinal preparation of large masses originating in the gut unless very necessary.

Organic disease occurs in 80–90% of adults with intussusception (16, 17), of which colorectal cancer is the primary cause (18). Emergency surgery is often forced due to the risk of strangulated intestinal obstruction and perforation. This can cause great physical and mental harm to a patient due to the disease itself or prophylactic ostomy (19).

A Japanese study on the elective laparoscopic investigation of patients with intussusception found that the mean age of patients was 69 (29–100) years, and the incidence due to cecal site tumors was 42.8%. One 93-year-old woman (BMI = 15.1) underwent laparoscopic exploration surgery following abdominal pain. During the operation, it was confirmed that cecal cancer had entered the colon, causing pain from splenic flexure of colon intussusception (2). In the current case study, a CT scan showed intussusception, and the ileocecal region was unclear (Figures 1, 2), suggesting that the mass had originated in this area. The absence of preoperative cancer markers and the imaging characteristics of the mass also suggested that it was more likely to be benign. Intraoperative findings from the current case study and prior study results indicate that an abnormal mesangium and ligaments in the ileocecal region and abnormal growth of an ileocecal mass are high-risk factors for intussusception. Thus, in cases of unexplained intussusception, the possibility that the tumor originated from the ileocecal region should be considered.

Enterogenous cysts are rare congenital malformations that were used by Harriman in 1958 to describe a neurogastrum, endoderm, or respiratory cyst (20). The most common site of enterogenous cysts is the spinal canal, which accounts for approximately 1% of tumors in this region (21). Enterogenous cysts occurring in the digestive tract are also more common in the jejunum and ileum and are often detected within 1 or 2 years of age due to obstructive symptoms, chronic abdominal pain, or an abdominal mass (22). The patient reported here grew into a healthy adult and sought medical attention for a cyst that had protruded from her anus. This finding indicated that the patient had not previously sought medical attention for symptoms caused by the cyst or that a congenital cyst in the ileocecal region had been detected during routine physical examination. Considering the rarity of this disease and the possibility of a malignant transformation (4), more awareness is needed to improve the rate of early diagnosis.

## 4 Conclusion

This report describes an extremely rare case of an adult patient with severe intussusception resulting from a large ileocecal enterogenous cyst combined with dysplasia. Ileocecal cyst intrusions into the entire colon, rectum, and out of the anus were observed. The study sought to provide sufficient clinical data and a literature summary required to improve awareness about this condition.

## Data availability statement

The original contributions presented in the study are included in the article/supplementary material, further inquiries can be directed to the corresponding author.

## Ethics statement

Written informed consent was obtained from the individual(s) for the publication of any potentially identifiable images or data included in this article.

## Author contributions

BZ: Conceptualization, Data curation, Funding acquisition, Writing – original draft, Writing – review & editing. XX: Investigation, Writing – review & editing. ND: Formal analysis, Writing – original draft. WW: Data curation, Software, Writing – review & editing. LP: Project administration, Software, Writing – review & editing. DF: Project administration, Software, Writing – review & editing. HW: Data curation, Project administration, Writing – review & editing. SH: Project administration, Validation, Writing – review & editing. ZL: Methodology, Supervision, Writing – review & editing. XZ: Conceptualization, Visualization, Writing – review & editing.
